# Changes in public attitudes towards confidential adolescent sexual and reproductive health services in Lithuania after the introduction of new legislation: findings from the cross-sectional surveys conducted in 2005 and 2012

**DOI:** 10.1186/s12913-015-1027-5

**Published:** 2015-09-04

**Authors:** Lina Jaruseviciene, Apolinaras Zaborskis, Skirmante Sauliune, Gediminas Jarusevicius, Jeffrey V. Lazarus

**Affiliations:** Department of Family Medicine, Lithuanian University of Health Sciences, Kaunas, Lithuania; Institute of Health Systems Research, Lithuanian University of Health Sciences, Kaunas, Lithuania; Department of Health Management, Lithuanian University of Health Sciences, Kaunas, Lithuania; Department of Cardiology, Lithuanian University of Health Sciences, Kaunas, Lithuania; Centre for Health and Infectious Disease Research (CHIP), Rigshospitalet, University of Copenhagen, Copenhagen, Denmark

## Abstract

**Background:**

In Lithuania, the right to confidentiality in healthcare for adolescents over the age of 16 was guaranteed in 2010 through the adoption of new legislation. This study sets out to explore changes in Lithuanian residents’ attitudes towards confidentiality protection in adolescent sexual and reproductive healthcare (SRH) by comparing data from surveys administered in 2005 and 2012.

**Methods:**

For both surveys, the participants were random samples of the Lithuanian residents aged 16 to 74. A 23-item questionnaire was used in 2005 and complemented with 2 items in 2012. Linear regression analysis was employed to estimate absolute differences in prevalence of belief in whether or not adolescents would find confidentiality important when consulting a physician on SRH issues. A log-binomial regression model was fitted to estimate the relative changes (prevalence ratio) of the independent variables.

**Results:**

The total number of respondents was 1054 (response rate 83 %) in 2005 and 1002 (response rate 80 %) in 2012. The proportion of respondents who reported a belief that adolescents would find confidentiality important when seeing a physician for SRH issues increased significantly from 62 % in 2005 to 73 % in 2012. Regardless of their belief in the importance of confidentiality, in 2012 respondents more often indicated positive outcomes on the relations between the physician and the minor patient, such as increased trust of the adolescent in the physician and more frequent visits to physicians. However, study participants who believed that adolescents would find confidentiality important in 2012 were less optimistic about potential positive outcomes of further legal consolidation of adolescents’ right to confidentiality than in 2005. Younger respondents were the most optimistic about potential outcomes if laws were enacted to further protect adolescent confidentiality.

**Conclusions:**

This study uncovers the dynamics of public attitudes towards the socially and ethically sensitive issue of adolescent SRH. Our study suggests that legislation could be a factor prompting changes in public opinion, but not sufficient in and of itself for its social acceptance.

**Electronic supplementary material:**

The online version of this article (doi:10.1186/s12913-015-1027-5) contains supplementary material, which is available to authorized users.

## Background

Confidentiality is one of the principal ethical standards that has shaped the nature of the physician-patient relationship since the Hippocratic epoch [1]. According to Beauchamp and Childress [2], the obligation to confidentiality in healthcare is justified by consequence, rights and fidelity-based arguments. A violation of confidentiality increases potential negative consequences because a lack of trust in the physician reduces the disclosure of information needed to make an appropriate medical decision. Regardless of these potential negative health consequences, a breach of confidentiality is primarily seen as a violation of personal autonomy and privacy. Moreover, confidentiality is an essential part of health professional ethics codes [2].

Studies in adolescent sexual and reproductive healthcare (ASRH) provide evidence supporting the importance of confidentiality. The existing body of research indicates that a lack of confidentiality decreases adolescents’ interest in seeing healthcare providers [3, 4], deteriorates physician-patient communication [5] and negatively affects continuity of care [6, 7]. Britto and colleagues [8] revealed that informational privacy was more important for adolescents than psychological, social and physical privacy. Confidentiality has been shown to be one of the leading reasons for adolescents to use preventive services [9] and is an important criterion of quality of care in general [10].

The United Nations Convention on the Rights of the Child stresses the necessity to consider the “evolving capacities” of minors, acknowledging parents’ duty to care for their children [11]. Legal limitations of parental power set in the convention strengthen adolescents’ decision-making capacities, encouraging them to foresee the consequences of their decisions and to take responsibilities for these decisions [12]. Protected by international law and professional consensus [13], an adolescent’s right to confidentiality, however, remains the object of persistent debate [14].

A qualitative study of Lithuanian primary health care physicians revealed that public opinion can be an important factor that influences medical decision-making in the context of adolescents’ needs, parents’ expectations, legal frameworks and organizational constraints [15]. Research performed in France indicated that the general public is more supportive of lax confidentiality as compared to medical professionals: breaching confidentiality in the event of a sexually transmitted infection (STI) of a sexual partner was considered as moderately acceptable while physicians perceived this as unacceptable behaviour [16]. Moreover, public attitudes towards confidentiality protection in ASRH might be at times contradictory or heterogeneous [17, 18]. For example, in a public opinion survey performed in 2005 in Lithuania, at least one-third of the respondents believed that in certain clinical situations related to ASRH, the doctor should guarantee confidentiality to those under the age of 16. At the same time, only 12 % were ready to support legalisation of the rights of those under 16 to confidential healthcare services [17].

Lithuania, a European Union member state since 2004, is still facing tremendous organizational and legal reforms, similar to other countries of the former Soviet Union [19]. In 1996 the Lithuanian Law on the Rights of Patients and Compensation for Health Damage was adopted [20]. Since then, several revisions of this law have come into force. Although previous legislation stressed an adolescent’s rights to confidentiality, legislation was ambiguous and parents or legal representatives had a right to access the medical information of patients younger than the age of 18. The most recent change came into force in March 2010, explicitly setting the age of confidentiality at 16. However, research indicate that legal provisions are largely unknown by the Lithuanian population. For instance, it was revealed that the existence of the Law on the Rights of Patients and Compensation for Health Damage was known by only 56 % of patients [21] and legal provisions concerning adolescent healthcare by 51 % of general practitioners [22].

The aim of this study was to explore the changes in Lithuanian residents’ attitudes towards confidentiality protection in ASRH by comparing data from two cross-sectional surveys. Specifically, we present: (i) the changes in public concern about the importance of confidentiality for adolescents; and (ii) the shifts in the public attitudes towards potential outcomes if laws are enacted to further protect adolescent confidentiality.

## Methods

A cross-sectional survey was conducted nationally in August 2005 [17] and again in April 2012, two years after the legal reform on the establishment of the right to confidentiality from the age of 16. Data from the Department of Statistics Lithuania and the Lithuanian population register were used to define the composition of all Lithuanian residents (not only Lithuanian nationals) in the country, based on their age, gender and administrative territory of their home location in order to perform a stratified population selection. For both surveys, random samples of the residents aged 16 to 74 representative by age, gender and administrative territory of their home location were created.

The Lithuanian market analysis and survey agency UAB RAIT was responsible for selecting the participants and conducting interviews. After verbal consent was obtained, respondents were interviewed in their households. Professional interviewers, using guidance provided to them by the research team, completed the questionnaires.

The Bioethics Committee of the Kaunas University of Medicine approved the study in 2005; in 2012 this committee determined that further ethical clearance to repeat the study was not needed.

A 23-item questionnaire on ASRH was used for the survey in 2005; it was supplemented with two items in 2012 (Additional file 1). The 2012 survey included the same items and questions along with two additional questions about respondents’ knowledge of current legal provisions concerning the age when minors are allowed to independently have confidential health services. This study focuses on three questions. Two of the questions were asked in both surveys. The first asked respondents to indicate their opinion on if adolescents would find confidentiality important when addressing physicians for sexual and reproductive healthcare issues (the answers “important” and “very important” were merged for the analysis). The second question asked respondents to anticipate up to three of eight possible outcomes if laws were enacted to further protect adolescent confidentiality in SRH consultations: (i) adolescents‘trust in physician would increase; (ii) adolescents would more often address physician; (iii) adolescents would be more inclined to disclose their problems to physicians; (iv) adolescents would stick to physicians recommendations more strictly; (v) the situation would not change; (vi) adolescents’ parents trust in physicians would decrease; (vii) adolescent − parents’ relationships would deteriorate; and (viii) teenagers would be more engaged in sexual activity.

The third question: “From what age according to current legislation do adolescents have the right to independently have confidential health services” was asked only in the 2012 survey. Respondents had to indicate this age themselves.

Data analysis was performed with the Statistical Package for Social Sciences (SPSS) version 21 (IBM Corporation, Somers, NY, USA) using descriptive statistics and chi squared and z tests to investigate group- and survey-specific differences for socio-demographic variables as well as the respondents’ attitudes towards the importance of confidentiality in adolescent sexual healthcare.

Survey respondents in 2005 and 2012 differed significantly from each other with regards to age, education, marital status, employment status, nationality and income. Data for the 2012 survey were adjusted for gender, age and marital status in accordance with the 2005 survey in order to represent the two groups of respondents equally in the pooled data sample.

Linear regression analysis was employed to estimate absolute differences in prevalence of belief as to whether or not adolescents would find confidentiality important during the physician’s consultation for sexual and reproductive health issues. Respondents’ gender, age, education, marital status, employment status, income, nationality, population of community of residence and survey year were included in the model as independent variables. The categories of the independent variables were transformed into indicator variables; the first category of each variable was selected as the reference category. The coefficients of the linear regression analysis denoted the difference in prevalence between each category and reference category.

In addition, the relative changes (prevalence ratio) of the independent variables were estimated. For this purpose, a log-binomial regression model was fitted. The model estimated the prevalence ratio (PR) between each tested category and the reference category. The interaction between indicator variables and survey year was tested but no significant trend interaction effects were detected.

The models were calibrated using the generalized linear models (GLM) procedure. The procedure used weighted least squares to estimate model parameters and the full-parameterization approach, with indicator (dummy) variables created for every category of a factor. The Wald method was adopted to calculate 95 % confidence intervals (CI) of estimations [23].

## Results

Of the 1,270 people selected for the 2005 survey, 124 were not home at the time of the survey and 92 declined to be surveyed. This resulted in 1054 study participants (83 % response rate). Of the 1,435 people selected for the 2012 survey, 174 were not home at the time of the survey and 259 declined to be surveyed. This resulted in 1,002 study participants (79.6 % response rate).

In both surveys, there were slightly more female respondents than male respondents and slightly more employed respondents than unemployed respondents. The majority of the respondents were married in both. Other socio-demographic characteristics are presented in Table 1. Non-participants did not differ significantly from survey respondents with regards to gender, age or administrative territory of their home location (data not show in table).

**Table 1:** Socio-demographic characteristics of participants, by survey year

	2005		2012 (crude)		2012 (adjusted)^a^		*p*-value^b^
	n	(%)	n	(%)	n	(%)	
*Total:*	1054	(100)	1002	(100)	1002	(100)	
*Gender:*							
Male	509	(48)	484	(48)	478	(48)	0.790
Female	545	(52)	518	(52)	524	(52)	
*Age (years):*							
16–34	331	(32)	329	(33)	314	(31)	0.998
35–54	393	(37)	305	(30)	373	(37)	
55–74	330	(31)	368	(37)	315	(32)	
*Marital status:*							
Married	603	(57)	455	(45)	565	(57)	0.540
Single	215	(20)	250	(25)	202	(20)	
Divorced	95	(9)	142	(14)	91	(9)	
Widow(−er)	101	(10)	116	(12)	92	(9)	
Unmarried cohabiting	38	(4)	39	(4)	52	(5)	
No response provided	2						
*Education:*							
Basic or less	212	(20)	207	(21)	185	(19)	**0.002**
General (secondary school)	338	(32)	292	(29)	293	(29)	
Further education	292	(28)	238	(24)	248	(25)	
Higher (university)	212	(20)	265	(26)	274	(27)	
*Employment:*							
Employed	562	(55)	462	(46)	520	(52)	0.170
Unemployed	461	(45)	540	(54)	482	(48)	
No response provided	31						
*Incomes (per month and family person):*							
Up to 500 Litas^c^	579	(61)	148	(18)	149	(18)	**<0.001**
501 to 1000 Litas	289	(30)	453	(55)	439	(53)	
More than 1000 Litas	89	(9)	227	(27)	239	(29)	
No response provided	97		175				
*Nationality:*							
Lithuanian	907	(87)	901	(90)	897	(90)	**0.048**
Other nationality	137	(13)	99	(10)	103	(10)	
No response provided	10		2				
*Population of community of residence:*							
Up to 2,000	346	(33)	327	(33)	324	(32)	0.74
2,000–180,000	352	(33)	349	(35)	350	(35)	
More than 180,000	356	(34)	326	(32)	327	(33)	

^a^Adjusted for gender, age and marital status in accordance with the survey in 2005

^b^Comparing 2005 and 2012 (adjusted) (Chi-squared test)

^c^1 Litas = €0.29

*p*-values in bold indicate a statistically significant difference

The 2005 and 2012 cohorts significantly differed in education level. In 2012, there were a larger proportion of former university students. The proportion of Lithuanian survey respondents (as opposed to other nationalities) increased from 86 % to 90 %. In 2005, the cohort included slightly more employed respondents than unemployed. In 2012, the participants included slightly more unemployed people. The proportion of respondents reporting an income of up to 500 litas per month declined from 61 % to 18 % while the proportions of respondents in the two higher income brackets (501–1,000 litas and more than 1,000 litas per month) increased.

### Perception of the importance of confidentiality for adolescents in sexual and reproductive healthcare

The proportion of respondents who indicated a belief that adolescents would find confidentiality important when addressing a physician for sexual and reproductive health issues increased from 61.9 % in 2005 to 73.0 % in 2012 (*p* < 0.001) (Table 2).

**Table 2:** Respondents who believed^a^ that adolescents would find it important that the physician would protect their confidentiality in a consultation on sexual and reproductive health issues, by survey year and socio-demographic characteristics

	2005 (*N* = 964)^b^	2012^c^ (*N* = 852)^b^	*p*-value^1^
	%	%	
*Total:*	61.9	73.0	**<0.001**
*Gender:*			
Male	58.6	70.4	**<0.001**
Female	65.0	75.1	**0.001**
p-value^2^	**0.042**	0.123	
*Age:*			
16–34	71.4	77.9	0.070
35–54	60.9	72.7	**0.001**
55–74	52.7	67.6	**<0.001**
p-value	**<0.001**	**0.029**	
*Education:*			
Basic or lower	56.7	68.7	**0.024**
General (secondary)	64.6	74.5	**0.012**
Further education	65.6	72.6	0.095
University	57.6	74.3	**<0.001**
p-value	0.241	0.743	
*Marital status:*			
Married	62.6	72.0	**0.002**
Single	70.4	77.8	0.097
Divorced	47.6	72.6	**0.001**
Widowed	50.0	69.0	**0.016**
Unmarried cohabiting	62.9	72.3	0.361
p-value	**0.001**	0.552	
*Employment status:*			
Employed	63.5	75.5	**<0.001**
Unemployed	60.0	70.2	**0.001**
p-value	0.284	0.082	
*Income (per month and family person)*			
Up to 500 litas^3^	62.5	74.4	**0.011**
501 to 1,000 litas	59.0	68.9	**0.010**
More than 1,000 litas	65.5	76.7	**0.050**
p-value	0.477	0.112	
*Nationality:*			
Lithuanian	62.3	72.6	**<0.001**
Other nationality	60.5	77.2	**0.014**
p-value	0.710	0.378	
*Population of community of residence:*			
Up to 2,000	64.3	68.7	0.147
2,000–180,000	63.5	73.2	**0.007**
More than 180,000	57.7	77.2	**<0.001**
p-value	0.179	0.072	

^a^These respondents indicated a belief that it is ‘very important’ or ‘important’ for adolescents to obtain confidentiality when addressing physician on sexual and reproductive health issues. Other response options were ‘neither important nor not important’, ‘not important’ and ‘not important at all’

^b^total number of respondents who provided required responses

^c^adjusted for gender, age and marital status in accordance with the survey in 2005

^1^comparing 2005 and 2012 within the same category (z test)

^2^comparing categories within the same year (Chi-squared test)

^3^1 litas = €0.29

*p*-values in bold indicate a statistically significant difference

The univariate analysis (data not shown) showed that the perception of the importance of confidentiality in both surveys was significantly related to a respondent’s age: younger respondents more often supported the notion that confidentiality is important for adolescents in SRH.

Linear regression demonstrated that females and younger age (16–34) were associated with the belief that confidentiality is important for adolescents consulting physicians about SRH issues (Table 3). It also showed that 2012 survey respondents were more likely than 2005 survey respondents to express this belief.

**Table 3:** Absolute and relative changes in proportion of respondents who believed^a^ that it was important to adolescents that the physician ensured confidentiality of their consultancy about SRH issues, by survey year^b^ and socio-demographic factors: results from the multivariate analysis

	Absolute change			Relative change		
	%	(95 % CI)	p-value	PR	(95 % CI)	p-value
Constant	63.1	(53.5 to 72.6)	**<0.001**	0.61	(0.53 to 0.71)	**<0.001**
*Gender:*						
Male (ref.)	0			1		
Female	6.9	(2.2 to 11.6)	**0.004**	1.12	(1.05 to 1.21)	**0.001**
*Age (years):*						
16–34 (ref.)	0			1		
35–54	−8.9	(−15.6 to −2.2)	**0.005**	0.88	(0.80 to 0.96)	**0.004**
55–74	−10.9	(−18.5 to −3.3)	**0.009**	0.85	(0.76 to 0.95)	**0.004**
*Education:*						
Basic or lower (ref.)	0			1		
General (secondary school)	7.1	(−0.1 to 14.3)	0.051	1.12	(1.00 to 1.25)	0.054
Further education	7.6	(−0.1 to 15.2)	0.051	1.13	(1.00 to 1.27)	0.057
Higher (university)	4.2	(−4.0 to 12.3)	0.317	1.08	(0.96 to 1.23)	0.198
*Marital status:*						
Married (ref.)	0			1		
Single	1.5	(−6.0 to 9.1)	0.688	1.03	(0.93 to 1.14)	0.572
Divorced	−4.4	(−12.1 to 3.2)	0.256	0.94	(0.84 to 1.06)	0.307
Widow(−er)	−5.2	(−13.5 to 3.1)	0.217	0.92	(0.80 to 1.06)	0.247
Family without a registered marriage	−2.5	(−14.0 to 9.1)	0.673	1.00	(0.85 to 1.17)	0.963
*Employment:*						
Employed (ref.)	0			1		
Unemployed	−3.0	(−8.8 to 2.7)	0.302	0.97	(0.89 to 1.05)	0.428
*Incomes (per month and family person)* ^*1*^						
Up to 500 litas	0			1		
501 to 1000 litas	−3.4	(−9.1 to 2.4)	0.249	0.94	(0.87 to 1.03)	0.174
More than 1000 litas	1.1	(−7.0 to 9.2)	0.793	1.00	(0.90 to 1.12)	0.992
*Nationality:*						
Lithuanian	0			1		
Other nationality	1.5	(−5.9 to 8.8)	0.699	1.03	(0.92 to 1.15)	0.634
*Size of location:*						
Up to 2,000 residents (ref.)	0			1		
2,000–180,000 residents	1.3	(−4.3 to 6.8)	0.659	1.04	(0.96 to 1.12)	0.375
More than 180,000 residents	−3.2	(−9.3 to 2.8)	0.296	0.97	(0.88 to 1.06)	0.481
*Survey year:*						
2005 (ref.)	0			1		
2012	11.3	(6.2 to 16.5)	**<0.001**	1.16	(1.08 to 1.25)	**<0.001**

PR: prevalence ratio

CI: Wald confidence interval

ref.: reference group

^a^who said that it is “very important” or “important” that physician ensures confidentiality of adolescent consultation

^b^data of surveys in 2005 and 2012 adjusted for gender, age and marital status

^1^1 litas = €0.29

*p*-values in bold indicate a statistically significant difference

### Outcomes anticipated by respondents if laws are enacted to further protect adolescent confidentiality

When assessing the outcomes anticipated by respondents if laws are enacted to further protect confidentiality in ASRH consultations, respondents were divided into two groups: respondents who indicated that adolescents would find confidentiality important and those who indicated that adolescents would not find confidentiality important (Table 4). Regardless of their belief in the importance of confidentiality, both groups more often indicated positive outcomes on the relations between the physician and the minor patient, such as increased trust of the adolescent in the physician or more frequent visits to physicians.

**Table 4:** Outcomes anticipated by respondents if laws are enacted to further protect adolescent confidentiality in sexual and reproductive health consultations, by survey year and respondent belief about the importance of confidentiality^a^

Outcomes	Adolescents would find confidentiality important^2^	Proportion of respondents who anticipated outcome		*p*-value^1^
		2005 (*N* = 964)^b^	2012^c^ (*N* = 852)^b^	
		%	%	
Adolescents’ trust in physicians would increase	Believed	61.8	51.0	**<0.001**
	Not believed	42.5	41.3	0.772
	p-value^2^	**<0.001**	**0.012**	
Adolescents would visit physicians more frequently	Believed	55.1	48.1	**0.014**
	Not believed	38.7	37.2	0.720
	p-value	**<0.001**	**0.005**	
Adolescents would be more inclined to disclose their problems to physicians	Believed	53.1	52.8	0.922
	Not believed	33.8	44.2	**0.011**
	p-value	**<0.001**	**0.025**	
Adolescents would follow physicians’ recommendations more strictly	Believed	19.4	22.1	0.258
	Not believed	12.5	14.3	0.524
	p-value	**0.005**	**0.012**	
Parents of adolescents would feel lees trusting of physicians	Believed	5.7	9.3	**0.016**
	Not believed	12.3	14.8	0.376
	p-value	**<0.001**	**0.023**	
Relationships between adolescents and their parents would deteriorate	Believed	6.4	7.2	0.542
	Not believed	11.7	10.9	0.751
	p-value	**0.004**	0.088	
Adolescents would be more likely to engage in sexual activity	Believed	3.4	6.0	**0.031**
	Not believed	9.3	7.4	0.426
	p-value	**<0.001**	0.446	
Situation would not change	Believed	13.2	9.8	0.062
	Not believed	23.2	19.0	0.234
	p-value	**<0.001**	**<0.001**	

^a^respondents were given the possibility to select up to three outcome options

^b^total number of respondents who provided the required responses

^c^adjusted for gender, age and marital status to match the 2005 survey

^1^comparing 2005 and 2012 (z test)

^2^comparing groups of respondents who indicated that adolescents would find confidentiality important and those who indicated that adolescents would not find confidentiality important when consulting on sexual and reproductive health issues (z test)

*p*-values in bold indicate a statistically significant difference

Regarding changes from 2005 to 2012, Table 4 shows that respondents who believed that adolescents would find confidentiality important in 2012 were less optimistic that legislative changes would lead adolescents to have greater trust in physicians (*p* < 0.001) or to visit physicians more frequently (*p* < 0.05). They also more often anticipated two negative effects of further legal consolidation of confidentiality guaranties in adolescent SRH: decreased trust of the parents of adolescents in physicians (*p* < 0.05) and more engagement of adolescents in sexual activity (*p* < 0.05). On the other hand, respondents who did not believe that adolescents would find confidentiality important in 2012, as compared to 2005, more often anticipated the positive effect of confidentiality: a higher predisposition of adolescents to disclose their problems to physicians when there were more confidentiality guarantees (*p* < 0.05) (Table 4).

The knowledge of legal provision concerning adolescents’ confidentiality protection was only assessed in the 2012 survey. The correct answer, i.e. that adolescents have the right to independently have confidential services from the age of 16, was provided by one third of the respondents (33.6 %), while 59.8 % indicated older than 16 years and 6.5 % younger than 16 years. There were no statistically significant differences between the responses by the respondents’ age group.

However, anticipated outcomes if laws were enacted to further protect adolescent confidentiality were statistically different for all three age groups (Table 5). The difference between the responses of age groups was mainly observed in the forecasting of positive outcomes of legal changes. Younger respondents (16–34 years old) were the most optimistic about potential outcomes. Forecasting how the potential legal changes would affect adolescents’ adherence to physicians recommendations, younger respondents in 2012 were even more optimistic than in 2005 (*p* < 0.01).

**Table 5:** Outcomes anticipated by respondents if laws are enacted to further protect adolescent confidentiality in sexual and reproductive health consultations, by survey year and respondent age group^a^

Survey year and Outcomes	Survey year	Proportion of respondents who anticipated outcome, by age group			p-value^1^
		16–34 years	35–54 years	55–74 years	
		%	%	%	
Adolescents’ trust in physicians would increase	2005	59.0	56.6	46.6	**0.006**
	2012	55.6	47.2	41.4	**0.004**
	p-value^2^	0.393	**0.014**	0.226	
Adolescents would visit physicians more frequently	2005	51.4	52.7	41.0	**0.007**
	2012	52.4	48.4	32.1	**<0.001**
	p-value	0.803	0.262	**0.035**	
Adolescents would be more inclined to disclose their problems to physicians	2005	53.3	47.8	34.6	**<0.001**
	2012	52.6	53.1	44.4	0.084
	p-value	0.863	0.166	**0.021**	
Adolescents would follow physicians’ recommendations more strictly	2005	15.9	17.8	16.6	0.802
	2012	25.5	18.0	16.4	**0.016**
	p-value	**0.003**	0.931	0.947	
Parents of adolescents would feel less trusting of physicians	2005	7.6	6.3	11.3	0.062
	2012	8.4	11.5	13.2	0.198
	p-value	0.727	**0.016**	0.515	
Relationships between adolescents and their parents would deteriorate	2005	6.7	8.2	10.6	0.220
	2012	9.5	7.7	7.4	0.636
	p-value	0.206	0.825	0.205	
Adolescents would be more likely to engage in sexual activity	2005	3.2	4.6	9.5	**0.002**
	2012	6.0	5.0	8.2	0.276
	p-value	0.100	0.843	0.599	
Situation would not change	2005	14.6	18.3	18.0	0.380
	2012	6.6	15.8	14.3	**0.001**
	p-value	**0.002**	0.382	0.255	

^a^respondents were given the possibility to select up to three outcome options

^1^ comparing age groups (chi squared test)

^2^comparing 2005 and 2012 year of the survey (z test)

*p*-values in bold indicate a statistically significant difference

## Discussion

This study, tracking the shift of concern for confidentiality in ASRH in Lithuania, found that residents there have attributed greater emphasis to the importance of confidentiality for adolescents in sexual and reproductive healthcare over time. There was a more positive perception of the importance of confidentiality for adolescents seeking sexual and reproductive health services in the 2012 survey as compared to the 2005 survey, and this held for all socio-demographic groups.

Although our study did not seek to detect determining factors for such a shift, it is very likely that the adoption of new legislation in 2010 played an important role. Previous surveys have underlined weak and uncertain knowledge of healthcare laws by different social groups not only in Lithuania, but in other countries as well [21, 22, 24, 25]. Our study, although not examining the legal knowledge of the study population, suggests that explicit legislation on the issue has had a favourable effect on its awareness in the general public.

These findings are in line with insights of the anthropologist M. Douglas [26], which revealed the core of the system of social relationships. Douglas used the metaphor of “purity and danger,” aiming to describe social classification. Because of the moral dimension in culture, activities, situations and events are categorised as socially right or wrong. A phenomenon that clearly contradicts existing classification systems or oversteps its limits disturbs the established order and is perceived by society as a social danger. A condition that does not have an exact place in the social classification system and cannot be attributed to being socially right nor socially wrong is placed in a “grey zone”. This non-attribution is implicitly perceived by society as “not pure” and, eventually, avoided by the society [26]. The confidentiality in ASRH belongs to the social and ethical “grey zone”. While the importance of confidentiality in healthcare in general is seldom questioned, confidentiality in adolescent healthcare often is. Thus, explicit Lithuanian legislation on adolescents’ rights to confidentiality in healthcare in 2010 [20] could be viewed as a placing of the phenomenon of confidentiality in adolescent healthcare in the system of social relationships, which contributes greatly to the positive dynamics of social attitudes.

However, the 2012 survey reported less optimistic anticipated outcomes in ASRH if laws are enacted to further protect the confidentiality of adolescent SRH. This might be the result of expectations that are too great regarding the further legislation of adolescent confidentiality among the 2005 survey participants. Further, the age of the adolescents that the respondents had in mind when anticipating the effects of future legalisation of confidentiality guaranties in 2005 and 2012 is not clear. Taking into account the recently decreased age of guaranteeing confidential services to 16, it is highly possible that in 2012 Lithuanian residents forecasted effects of improved confidentiality protections for adolescents under 16 years of age, while in 2005 they could have had in mind adolescents younger than 18. The fact that 40 % of the respondents in 2012 indicated 16 years or younger as an age from which an adolescent has the right to confidential health services would support this insight. However, the legal knowledge of the population was not assessed in the 2005 survey making comparisons impossible. A decrease in optimism in the forecasting of outcomes of future legal legislation of confidentiality in adolescent healthcare was observed only in older age groups. Younger respondents in both surveys demonstrated a consistently positive attitude.

A greater understanding of the importance of confidentiality for adolescents in sexual and reproductive healthcare combined with increased concerns about its potentially negative outcomes suggests that public attitudes concerning adolescent confidentiality is a complex phenomenon. This apparent inconsistency in beliefs was also found in other studies. For example, the authors of a study addressing parents’ support for confidentiality in adolescent healthcare in the USA [14] reported that answers of respondents became more conservative when the word “law” was in the question. Moreover, they suggest that parents recognizing the importance of confidentiality on an intellectual level might disagree with the legalisation of a minor’s right to confidential health services as they want to control their children, seeing this as their role as a parent [14]. This could suggest that legislative changes alone are not sufficient for the full social acceptance of the right to confidentiality in ASRH. Thus, specific public education campaigns coupled with interventions targeted at adolescents’ parents or guardians in healthcare settings could be instrumental in eliminating fears that are potentially related to confidentiality in ASRH and in shaping a new understanding of parenthood.

This study has several limitations including that the sample structure of the two surveys is different. Among the 2012 study participants, there were more Lithuanians, wealthy and educated people as compared to the 2005 survey. In spite of the natural socio-demographic changes in Lithuanian society that could explain the differences in the two samples, we adjusted the data of the 2012 survey for gender, age and marital status to match the survey in 2005. Another limitation is related to the study design. Both studies were cross-sectional, which does not allow for demonstrating the causality of the changes observed.

When developing policies on ethically and socially sensitive issues public opinion surveys are often invoked as a weighty argument [27–30]. In spite of the aforementioned limitations, this study suggests that legislation itself could be a factor prompting changes in public opinion. In a democratic political system, legislation of an issue is often seen as the institutionalization of the public’s will.

## Conclusions

This study contributes to the understanding of the dynamic of public attitudes towards the socially and ethically sensitive issue of adolescent sexual and reproductive healthcare. It suggests that legislation could be a factor prompting changes in public opinion, but not sufficient in and of itself for its acceptance among the population. Public information campaigns coupled with interventions targeted at adolescents’ parents in healthcare settings could be instrumental in eliminating fears that are potentially related to confidentiality in adolescent sexual and reproductive healthcare.

## Additional file

Additional file 1:
**Questionnaire.** (DOCX 30 kb)
