# Supplementary material for: Changes in public attitudes towards confidential adolescent sexual and reproductive health services in Lithuania after the introduction of new legislation: findings from the cross-sectional surveys conducted in 2005 and 2012
Source: BMC Health Serv Res. 2015 Sep 4;15:360. doi: 10.1186/s12913-015-1027-5 (PMC4560918; doi:10.1186/s12913-015-1027-5)
Supplement: Additional file 1: — Questionnaire. (DOCX 30 kb) [file 12913_2015_1027_MOESM1_ESM.docx]

**Questionnaire**

1. ***What is your gender?***

□ Male

□ Female

□Not indicated

1. ***What is your age?***

□ 16-24 years

□ 25-34 years

□ 35-44 years

□ 45-54 years

□ 55-64 years

□ 65-74 years

□Not indicated

1. ***What is your marrital status?***

□ Married

□ Single

□ Divorced

□ Widowed

□ Unmarried cohabiting

□Not indicated

1. ***What is your education?***

□ Basic or lower

□ General (secondary school)

□ Further ( post-secondary education:college, vocational training)

□ Higher (university)

□Not indicated

1. ***Are you currently employed?***

□ Yes

□ No

□Not indicated

1. ***What is the income per one of your family person per month?***

| □ Up to 500 Litas |
| --- |
| □ 501 to 1000 Litas |
| □ 1001 – 2000 Litas □ 2001-3000 Litas  □ 3001-4000 Litas  □ More than 4001 Litas  □Not indicated   1. ***What is your nationality?***   □ Lithuanian  □ Other  □Not indicated   1. ***What is your county of the residence?***   □ Vilniaus  □ Klaipėdos  □ Kauno  □ Alytaus  □ Tauragės  □ Šiaulių  □ Utenos  □ Telšių  □ Panevėžio  □ Marijampolės   1. ***What is the size of the population of your community of residence?***   □ Up 2000  □ 2000- 30000  □ 30000 – 100000  □ 100000- 180000  □ More than 180000   1. ***Have you ever seen in your health care institution the written information related to protection of medical secrecy (confidentiality) for minor patients (adolescents)?***   □ Yes  □ No  □ Do not know   1. ***Can parents get the medical records of their minor children at the reception of your health care institution?***   □ Yes  □ No  □ Don’t know   1. ***According to your opinion, how much adolescents would find confidentiality important when addressing a physician for sexual and reproductive health issues?***   □ Very much important → question No13  □ Important → question No13  □ Neither important, nor not important → question No14  □ Not important → question No14  □ Not important at all → question No14   1. ***According to your opinion, what kind of changes would be the most useful when improving confidentiality of sexual and reproductive healthcare services provided to adolescents?*** *(please select up to 3 options)*   □ Adoption of legislation which clearly regulate physician’s tactics of keeping medical secret of minors’ consultation  □ Development of the recommendations on confidentiality in adolescent healthcare by the professional and academic medical organizations  □ Development of written office policy concerning the confidentiality protection in healthcare institutions  □ Provision of information about importance of confidentiality to the minors in the waiting rooms, registration desks, etc.  □ Implementation of more strict order of keeping and dispensing the medical records to the patients and their representatives  □ Improvement of the privacy of physician and patient consultations (other persons, including medical staff, like a nurse, should not participate at the consultation)  □ Provision of more information about confidentiality in adolescent sexual healthcare in mass media  □ Don‘t know   1. ***How, according to your opinion, physician should behave in consulting 14-17 years old adolescents on sexual and reproductive health issues? (one option)***   □ Always to disclose to parents (guardians) the content of consultation  □ To inform parents (guardians) only in case, if he meets him  □ To inform parents (guardians) only in case if they directly ask physician about the consultation  □ To disclose the content of consultation to parents (guardians) only in exceptional cases  □ Never to disclose the content of consultation to adolescent parent (guardian)  □ Don’t know   1. ***According to your opinion, physician should maintain the confidentiality of 14-15 year old adolescent consultation and not to disclose to his parents (guardians) that he (she)is*** *(please select one answer in each row)****:***  \|  \| **Trully**  **yes** \| **Yes** \| **Neither yes,  nor no** \| **No** \| **Trully**  **no** \| \| --- \| --- \| --- \| --- \| --- \| --- \| \| 1. Interested in sexual issues \| □ \| □ \| □ \| □ \| □ \| \| 2. Had begun sexual relationships \| □ \| □ \| □ \| □ \| □ \| \| 3. Use condoms or other contraception \| □ \| □ \| □ \| □ \| □ \| \| 4. Has sexually transmitted infection, not consent for  treatment \| □ \| □ \| □ \| □ \| □ \| \| 5. Has sexually transmitted infection, consent for treatment \| □ \| □ \| □ \| □ \| □ \| \| 6. Is pregnant (or the girlfriend is pregnant) \| □ \| □ \| □ \| □ \| □ \| \| 7. Request abortion \| □ \| □ \| □ \| □ \| □ \| \| 8. Disclose his(her) homosexual experience \| □ \| □ \| □ \| □ \| □ \|   16. ***According to your opinion, physician should maintain the confidentiality of 16-17 year old adolescent consultation and not to disclose to his parents (guardians) that he (she)is*** *(please select one answer in each row)****:***   \|  \| **Trully**  **yes** \| **Yes** \| **Neither yes,  nor no** \| **No** \| **Trully**  **no** \| \| --- \| --- \| --- \| --- \| --- \| --- \| \| 1. Interested in sexual issues \| □ \| □ \| □ \| □ \| □ \| \| 2. Had begun sexual relationships \| □ \| □ \| □ \| □ \| □ \| \| 3. Use condoms or other contraception \| □ \| □ \| □ \| □ \| □ \| \| 4. Has sexually transmitted infection, not consent for  treatment \| □ \| □ \| □ \| □ \| □ \| \| 5. Has sexually transmitted infection, consent for treatment \| □ \| □ \| □ \| □ \| □ \| \| 6. Is pregnant (or the girlfriend is pregnant) \| □ \| □ \| □ \| □ \| □ \| \| 7. Request abortion \| □ \| □ \| □ \| □ \| □ \| \| 8. Disclose his(her) homosexual experience \| □ \| □ \| □ \| □ \| □ \|  1. ***Starting what age, according to your opinion, adolescents should have the right to address physicians independently?***   Please indicate....   1. ***Starting what age, according to your opinion, adolescents should have the right to independently have confidential health care services?***   Please indicate....   1. ***Starting what age, according to existing legal basis, adolescents have right to address physician independently?*** *(only in 2012 survey)*   Please indicate....   1. ***Starting what age, according to existing legal basis, adolescents have right to independently have confidential health care services?***  *(only in 2012 survey)*   Please indicate....   1. ***What outcomes you would anticipate if laws are enacted to further protect adolescent confidentiality in sexual and reproductive health consultations?*** *(please select up to 3 options)*   □ Adolescents’ trust in physicians would increase  □ Adolescents would visit physicians more frequently  □ Adolescents would be more inclined to disclose their problems to physicians  □ Adolescents would follow physicians’ recommendations more strictly  □ Parents of adolescents would feel lees trusting of physicians  □ Relationships between adolescents and their parents would deteriorate |
| □ Adolescents would be more likely to engage in sexual activity |
| □ Situation would not change   1. ***What measures, according to your opinion, would have the most favourable effect on the decrease of pregnancies and sexually transmitted infections among adolescents?*** *(please select one answer in each row)* |
|  |
|  |
| \|  \| **Trully**  **yes** \| **Yes** \| **Neither yes,  nor no** \| **No** \| **Trully  no** \| \| --- \| --- \| --- \| --- \| --- \| --- \| \| 1. Improve the sexual education in families \| □ \| □ \| □ \| □ \| □ \| \| 2. Strenghen the moral education in families \| □ \| □ \| □ \| □ \| □ \| \| 3. To promote abstinence during sexual education in schools \| □ \| □ \| □ \| □ \| □ \| \| 4. To provide information about contraception and sexually transmitted infection suring sexual education in schools \| □ \| □ \| □ \| □ \| □ \| \| 5. To facilitate the aquisition of contraceptive measures for sexually active young people (decreasing price, expanding points of sale, etc) \| □ \| □ \| □ \| □ \| □ \| \| 6. To facilitate young peoples‘ access to physicians – sexual and reproductive health care specialists, omitting the requirement to have referral from general practitioner \| □ \| □ \| □ \| □ \| □ \| \| 7. To open more youth health centers where young people could address freely and receive professional aid on sexual health issues \| □ \| □ \| □ \| □ \| □ \| |
| 1. ***What specialist, according to your opinion, would be the best counsellor on sexual and reproductive health issues for people younger than 18 years? (please select one option)***   □ General practitioner  □ Obstetrician gynecologist  □ Urologist  □ Dermatovenerologist  □ Physician, working in youth health center  □ Psychologist  □ Public health specialist (school nurse)  □ Don’t know   1. ***How would you evaluate sexual health services currently provided for people younger than 18 years?***   □ Very good  □ Good  □ Nor good, neither bad  □ Bad  □ Very bad   1. ***How would you evaluate sexual education currenly provided in schools?***   □ Very good  □ Good  □ Nor good, neither bad  □ Bad  □ Very bad |
